# Investigating the Underlying Effect of Thermal Modification on Shrinkage Behavior of Bamboo Culm by Experimental and Numerical Methods

**DOI:** 10.3390/ma14040974

**Published:** 2021-02-19

**Authors:** Raviduth Ramful, Thefye P. M. Sunthar, Wenliang Zhu, Giuseppe Pezzotti

**Affiliations:** 1 Graduate School of Science and Technology, Kyoto Institute of Technology (KIT), Matsugasaki, Sakyo-ku, Kyoto 606-8585, Japan; 2 Mechanical and Production Engineering Department, Faculty of Engineering, University of Mauritius, Reduit 80837, Mauritius; 3 Ceramic Physics Laboratory, Kyoto Institute of Technology, Matsugasaki, Sakyo-ku, Kyoto 606-8585, Japan; d0871502@edu.kit.ac.jp (T.P.M.S.); wlzhu@kit.ac.jp (W.Z.); pezzotti@kit.ac.jp (G.P.); 4 Department of Immunology, Graduate School of Medical Science, Kyoto Prefectural University of Medicine, Kamigyo-ku, 465 Kajii-cho, Kawaramachi Dori, Kyoto 602-0841, Japan; 5 The Center for Advanced Medical Engineering and Informatics, Osaka University, Yamadaoka, Suita, Osaka 565-0871, Japan; 6 Department of Orthopedic Surgery, Tokyo Medical University, Tokyo 105-8461, Japan

**Keywords:** bamboo, thermal modification, thermal gradient, thermal contraction, shrinkage, longitudinal split, FEM (finite element method)

## Abstract

This study probes into the root cause of split in thermally modified bamboo culm by investigating the underlying effect of thermal contraction with respect to its orthotropic nature by experimental and numerical methods while concurrently monitoring the chemical variation of its structure by Fourier transformed infrared spectroscopy (FTIR). In first part of this study, a non-linear increase in dimensional and weight changes of small clear bamboo specimens were observed with increasing temperature. The dimensional changes in the radial and tangential directions significantly exceeded that in the longitudinal direction. From FTIR results, shrinkage effect between 150 °C to 200 °C was associated with weight loss engendered by reduction in weakly bound water and increase in desorption of water content while alteration of its mechanical properties was attributed to changes in cellulose, hemicellulose, and lignin. From results of finite element method (FEM), the graded variation in thermal expansion coefficient, which showed the formation of a narrowed region of strain concentration corresponding to longitudinal crack propagation, was associated with the inducement of internal forces, namely tensile and compressive forces, at specific regions along the culm length. The results of this study can be useful to achieve optimized durability in modified bamboo for construction.

## 1. Introduction

The use of bamboo as a construction material in buildings dates back to thousands of years. It has a formidable strength to weight ratio with respect to wooden elements. Being a sustainable material, it has attracted much interest in modern eco-friendly design in recent years and has the advantage of reducing the dependency on conventional timber products. Additionally, the harvest cycle of mature bamboo is half than that of the fastest growing softwood at around 4 to 5 years. These important characteristics repositioned bamboo as an alternative consideration to conventional construction materials such as steel and concrete as it is considered to be a viable structural material such as trusses in construction given its low cost and energy saving benefits [1,2,3]. However, being a kind of high anisotropic biomaterial, bamboo displays a complex fracture behavior in various modes of loading [4,5,6,7,8,9]. Besides, changes in physical characteristics, such as in its moisture content often results in fracture along culm length in terms of longitudinal split. Rapid changes in relative humidity in the atmosphere and uneven drying during thermal modification are main causes of split in bamboo culm [10].

Pronounced cracking engendered by inhomogeneous shrinkage and swelling processes was the predominant damage mechanisms observed in large-timber structures in various climatic conditions [11]. Bamboo has high hygroscopicity due to the presence of polar hydroxyl groups in its cellulose fibers [12]. It will absorb or desorb moisture to attain equilibrium moisture content (EMC) equivalent to the ambient humidity and temperature [13]. In hygroexpansion-related investigation, the swelling and shrinkage of bamboo was found to be related to an increase and decrease in relative humidity (RH), respectively [14]. The hygroscopicity of bamboo is susceptible to change when exposed to heat. An increase in temperature and time exposure was found to affect the hygroscopic property of bamboo [15]. Natural shrinkage occurs as from room temperature, at 30 °C whereby bamboo loses free water deposited in its cell cavities [10]. During heat treatment, further shrinkage occurs as bamboo loses all free water up to 130 °C and absorbed water in cell walls between 180 °C and 220 °C [10]. In previous studies, the ductile characteristics of bamboo were reported to increase with increasing moisture content (MC) below the fiber saturation point (FSP) [16,17,18]. Furthermore, the mechanical properties of lignin-hemicellulose matrix were found to be more sensitive to changes in MC than cellulose [17]. However, the temperature in heat treatment exceeding 200 °C demonstrated causing a decrease in modulus of elasticity [10,19,20]. Harvested bamboo needs to be dried to reduce the moisture content to prevent degradation by insects and fungi. The drying of bamboo requires specific conditions of temperature and duration in order to prevent cracking [21]. To reduce the swelling and shrinkage in bamboo, several studies have considered heat treatment modification to improve its dimensional stability [19,20,22]. As treatment temperature increased, this led to a decrease in free hydroxyl groups in bamboo thereby reducing its hygroscopicity. This resulted in an improved anti-shrinking efficiency of heat-treated bamboo [20]. Furthermore, bamboo heat-treated in oil displayed improved hydrophobic characteristics and better dimensional stability as compared to simply heat-treated ones [20].

The splitting of bamboo varies among species as it depends on morphological features such as node interspacing, culm diameter and wall thickness. The structural role of the node in the culm affects the splitting in bamboo due to change in orientation of fibers in the vicinity of the nodes [23]. Given its unique morphology, bamboo has developed into a functionally graded material (FGM) structure transversely with smaller and denser vascular bundles in its outer-wall section and larger but fewer ones in its inner-wall section [24]. The transport of waterborne nutrients mainly takes place via its longitudinal system of metaxylem vessels running through the culm, which are connected to surrounding varying volume fraction of parenchyma and fibers [25,26]. Besides, the absorption of water radially is considerably restricted by the presence of high silica content in the outer layer, and absence of ray cells [27,28]. Inhomogeneous shrinkage was found to arise from uneven moisture distribution and orthotropic properties of wood which gave rise to moisture-induced stresses in the material [13]. In an investigation concerning the effect of heat on shrinkage of bamboo, the longitudinal shrinkage was found to be negligible as compared to radial and tangential shrinkage [15]. One of the previous studies showed that the hygroexpansion of bamboo in the longitudinal direction was smaller as compared to radial and tangential direction [14]. Tangentially, the outermost layers of dense fibers displayed greater shrinkage in comparison to innermost layer as a result of the inherent FGM structure which prevails transversely [20]. In the radial direction, non-uniform swelling was found to occur as outermost layers consisting of higher volume fraction of fibers tend to absorb more water than the innermost layers [15].

The investigation about shrinkage of bamboo is not well documented in the literature. Even though finite element method (FEM) has been widely applied in the investigation of macro-structural behavior of bamboo culms [29,30,31,32,33,34,35], its use to analyze crack growth emanating from climatic variations in such material remains unavailable to this date. Few studies have used the heat transfer analysis in FEM to analyze the effect of change in MC given the similarity which prevails between heat transfer and moisture transfer [13]. This study is focused on the investigation of shrinkage resulting from thermal effect on the fracture mechanisms of bamboo culm. In first part of this study, the shrinkage with respect to bamboo orthotropic nature was determined via experimental investigation of small clear specimens. The shrinkage by thermal contraction was conducted by heating inside oven and the weight loss and dimensional changes were recorded. The heating range was determined from the thermal gravimetric analysis (TGA) curve. Changes to its cellular structure due to thermal contraction are given by FTIR analysis. In second part of this study, the fracture mechanisms in full-culm bamboo structure due to shrinkage was modelled by FEM by considering variation in gradient of thermal expansion coefficient across its wall thickness.

## 2. Materials and Methods

### 2.1. Specimen Preparation and Pre-Treatment

**Figure 1:**
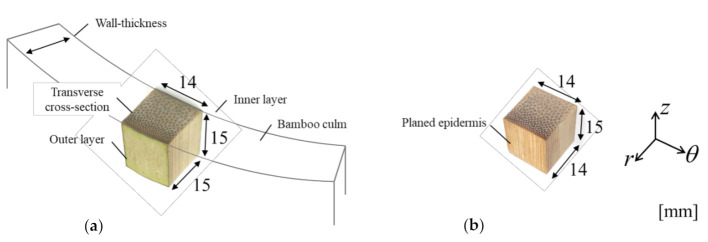
Schematic illustration of specimen sizing: (**a**) with epidermis and (**b**) with planed epidermis.

Bamboo specimens were prepared from untreated Moso bamboo (*Phyllostachys edulis*) obtained from Kyoto, Japan with maturity of 2 years. All specimens were cut from same internodal section as illustrated in Figure 1 to minimize microstructural variation at different length scales. The section of Moso bamboo used in this study had an approximate culm diameter of 15 cm and a wall thickness of 1.5 cm.

In the first batch, cubed specimens, with their outer epidermis removed, had average dimensions of 15 mm (longitudinal) × 14 mm (tangential) × 14 mm (radial). A second batch of bamboo specimens similar to the cubed dimensions was considered in the investigation by taking into account their outermost epidermis layer. This batch of cubed specimens was sized based on full wall thickness and had average dimensions of 15 mm (longitudinal) × 14 mm (tangential) × 15 mm (radial). A total of 10 specimens were investigated in each batch A and batch B.

The prepared specimens were pre-treated and conditioned prior to data measurement in shrinkage test. All specimens were dried at ambient temperature and humidity of 25 °C and 60 % inside laboratory environment for 1 week followed by oven dry for 72 h at 60 °C. The dried specimens were stored in a desiccator with humidity absorber for 1 week to absorb remaining residual moisture content. Hours before the shrinkage test, the middle section of bamboo specimen in each batch were measured with a digital Vernier caliper with an accuracy of ±0.02 mm.

### 2.2. Determination of Heating Range

The heating range and temperature intervals were determined by conducting Thermal Gravimetric Analysis (TGA). The TGA curves and the first derivative of the TGA curve, the differential thermo-gravimetric (DTG) curve, were obtained by a TGA machine (Discovery TGA, TA Instruments, New Castle, DE, USA) with a heating rate of 10 C/min in air atmosphere. The change in mass was measured by the Tru-Mass balance, a sensitive and accurate thermobalance inside the TGA equipment, which has an auto-ranging capability to deliver accurate real-time weight data. The solid curve represents the TGA results of Moso bamboo and its corresponding weight loss percentage with temperature while the dotted curve shows the corresponding weight loss with time (derivative).

**Figure 2:**
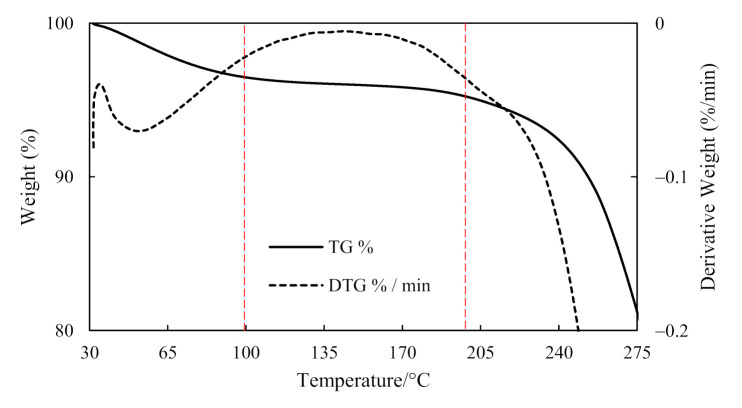
TGA test results of Moso bamboo to determine heating range.

Modification and changes in the composition of the main components such as cellulose, hemicelluloses and lignin have been reported in heat-treated wood [36,37]. The chemical constituents of bamboo which is similar to wood [38] undergoes major changes in terms of decomposition at elevated temperature above 200 °C [15]. Even so, the decomposition of cellulose in comparison to the other components has been reported to be lower due to its crystalline structure [39]. From the TGA results of Figure 2, the degradation of bamboo in terms of weight change is observed to start at about 150 °C. As this study investigates the thermal effect on shrinkage of bamboo, the temperature range within the TG plateau prior to degradation was considered. Hence, in addition to ambient temperature of 25 °C, the other set of temperature values investigated based on the TG plateau was 100 °C, 150 °C and 200 °C.

### 2.3. Experimental Procedure for Shrinkage Test

**Figure 3:**
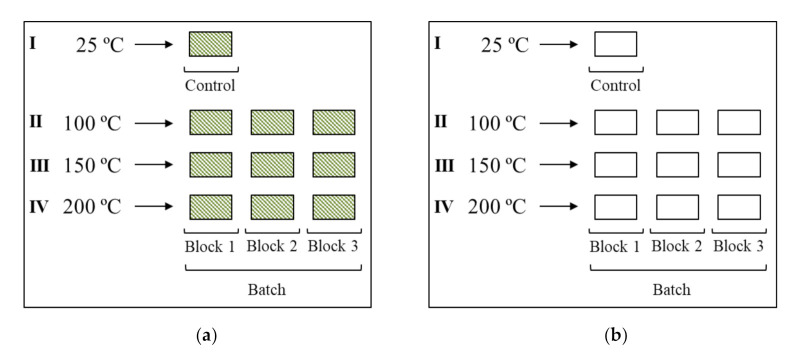
Test outline showing batch grouping of specimen: (**a**) with epidermis and (**b**) with planed epidermis.

As per the test outline in Figure 3, the specimens were grouped in sets of 3 and were subjected to three different temperatures, namely 100 °C, 150 °C, and 200 °C for a 24- and 48-h duration. The changes in weight and dimensions of the heat-treated specimens were subsequently recorded.

### 2.4. FTIR Analysis

The effect of thermal modification on the cellular constituents of bamboo was assessed from FTIR spectroscopy. FTIR analysis was performed by attenuated total reflection Fourier transform infrared spectroscopy (ATR-FTIR, FTIR-4700 with ATR PRO ONE fitted with a diamond prism; Jasco Co., Tokyo, Japan) which had a resolution of 4 cm^−1^ and including 100 scans. Spectral acquisition and pre-processing of raw data by baseline subtraction, smoothing, normalization and fitting methods were conducted in commercial software (Origin 8.5, OriginLab Co., Northampton, MA, USA, and LabSpec, Horiba/Jobin-Yvon, Kyoto, Japan).

## 3. Experimental Results

### 3.1. Effect of Thermal Modification on Physical Changes

**Figure 4:**
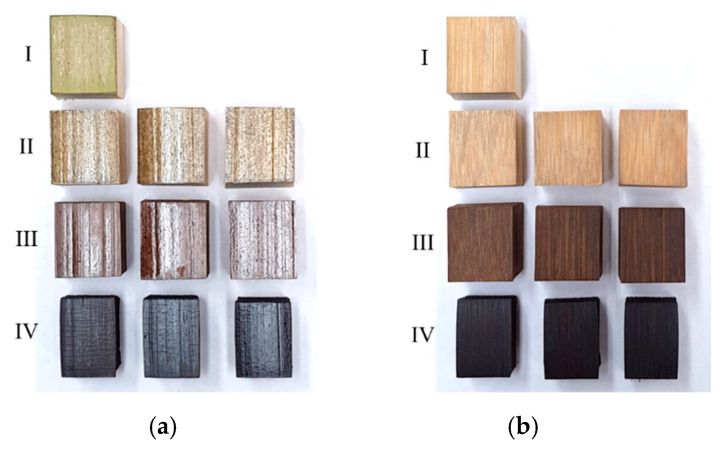
Visual changes following heat treatment in test specimen: (**a**) with epidermis and (**b**) with planed epidermis. Stages I, II, III and IV correspond to heat treatment at 25 °C, 100 °C, 150 °C and 200 °C, respectively, following a 48-h duration.

The visual changes in test specimens subjected heat treatment at 100 °C, 150 °C and 200 °C following a 48-h duration could be clearly discerned as displayed in Figure 4. The black color in stage IV correspond to partial carbonization of bamboo specimen at 200 °C.

**Figure 5:**
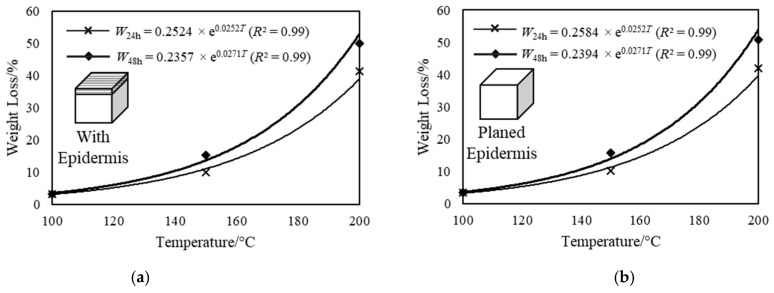
The percentage weight loss (including *R*^2^ values) of test specimen: (**a**) with epidermis and (**b**) with planed epidermis subjected to heat treatment at three different temperatures for 24- and 48-h duration.

The following observations in weight loss were made during the shrinkage experiment. A non-linear increase in weight loss was observed with increasing temperature. The exponential function, which is used to represent the trend line in Figure 5 and relates the weight loss to temperature, is given by:

*W* = Ae^B*T*^ (1)

where *W* is the weight loss (%), *T* is the temperature (°C) and A and B represents the coefficients obtained by curve fitting. The rate of weight loss significantly increased after 150 °C. Only slight increase in weight loss was observed between the 24-h to 48-h treatment period as compared to the first 24-h treatment period. The mean and coefficient of variation (COV) of weight loss in 24- and 48-h treatment periods are given in Table 1.

**Figure 6:**
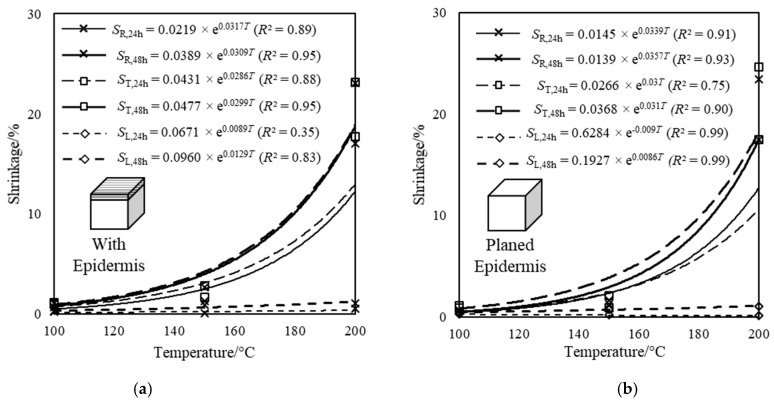
The dimensional changes due to shrinkage (including *R*^2^ values) in radial, tangential and longitudinal directions of test specimen: (**a**) with epidermis and (**b**) with planed epidermis subjected to heat treatment at three different temperatures for 24- and 48-h duration.

The following observations in terms of dimensional changes in the radial, tangential, and longitudinal directions were made during the shrinkage experiment. Similar to the observation made in weight loss, a non-linear increase in shrinkage principally occurred in radial and tangential dimensions with increasing temperature, for which the rate significantly increased after 150 °C as shown in Figure 6. The shrinkage in the radial and tangential directions considerably exceeded the shrinkage in the longitudinal direction. This difference further increased with increasing temperature.

The ratio of shrinkage in the radial and tangential directions to that in longitudinal direction significantly increased with temperature. Only slight increase in shrinkage was observed between the 24-h to 48-h treatment period. It can be deduced that substantial shrinkage occurred within the first 24-h treatment period. Secondly, the shrinkage difference between specimens with and without epidermis layer in all three directions was found to be negligible. The mean and coefficient of variation (COV) of dimensional changes in 24- and 48-h treatment periods are given in Table 1.

### 3.2. FTIR Analysis

**Figure 7:**
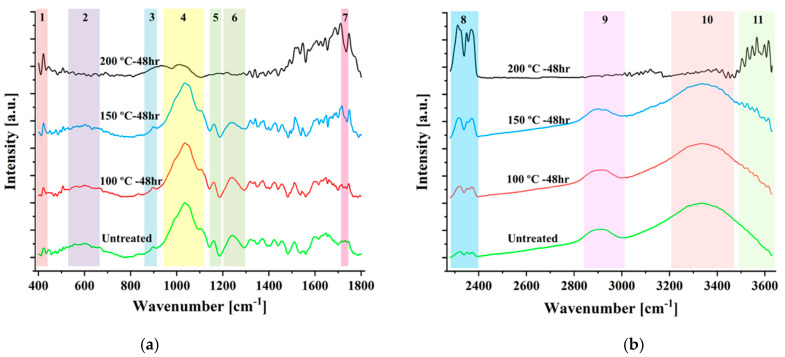
FTIR spectra of untreated and thermally modified bamboo at three different temperatures for a 48-h duration in two specific ranges, namely: (**a**) 400–1800 cm^−1^ and (**b**) 2280–3600 cm^−1^.

The effect of thermal modification on the main cellular constituent of bamboo such as cellulose, hemicellulose and lignin was qualitatively assessed by relative comparison of FTIR spectra. Two specific ranges of the spectrum were considered, namely 400–1800 cm^−1^ and 2280–3600 cm^−1^ and any distinct changes in peaks of FTIR spectra were highlighted and numbered accordingly as shown in Figure 7.

Cellulose was stable until 150 °C and degraded with further increase in temperature toward 200 °C as indicated by the decrease in intensity of peaks 3 (897 cm^−1^) and 9 (2945 cm^−1^) which contribute to the cellulose molecule in bamboo as per the assignment shown in Table 2. Similar trend in terms of intensity of peaks 4 (1039 cm^−1^) and 6 (1242 cm^−1^) which correspond to high proportion of guaiacyl units in lignin molecule of bamboo was observed up to 150 °C followed by their considerable reduction toward 200 °C. Notable degradation in hemicellulose was also demonstrated by the flattened peaks 5 (1160 cm^−1^), 6 (1242 cm^−1^), 7 (1730 cm^−1^) and 9 (2945 cm^−1^) at 200 °C which are associated with hemicellulose molecule in bamboo as per the assignment in Table 2. Based on the trend in degradation among the main chemical constituents of bamboo as observed with increasing temperature between the range of 150 °C to 200 °C, thermal modification within that range severely compromised its mechanical characteristics. The mechanical properties are predominantly governed by cellulose and lignin components which accounts for 44 and 20 %, respectively in terms of chemical constituent of bamboo [23].

Additionally, the reduction in weight during thermal modification at 200 °C occurs as a result of condensation reaction which lowers the amount of OH groups as shown by the decrease in intensity of peak 10 (3400 cm^−1^) which is linked to weakly bounded absorbed water as per the assignment in Table 2 [41]. Furthermore, as reported in previous investigation, increase in amount of adsorbed carbon dioxide, as evidenced by peak 8 (2320–2370 cm^−1^), also results in an increase in desorption of water content [46]. Interestingly, color change with increasing temperature as shown in Figure 4, occurs due to the formation of new chromophores such as carbonyl and carboxyl groups as a result of bond cleavage and subsequent oxidation and dehydration in polysaccharides. The high number of extractives responsible for color change occurs with higher temperature leading to cleavage in lignin bond and methoxyl groups as evidenced by peaks 4 (1039 cm^−1^), 10 (3400 cm^−1^) and 11 (3560–3620 cm^−1^).

## 4. Investigation of Thermal Contraction by FEM

### 4.1. Shrinkage Modeling

Here, the fracture mechanisms in full-culm bamboo structure due to shrinkage were modeled by FEM. The effective stress or Von-Mises stress have previously been used to investigate the deformation mechanisms in numerically simulated bamboo models [30,48,49]. In this study, the effective strain distribution was considered to investigate the deformation due to thermal contraction.

**Figure 8:**
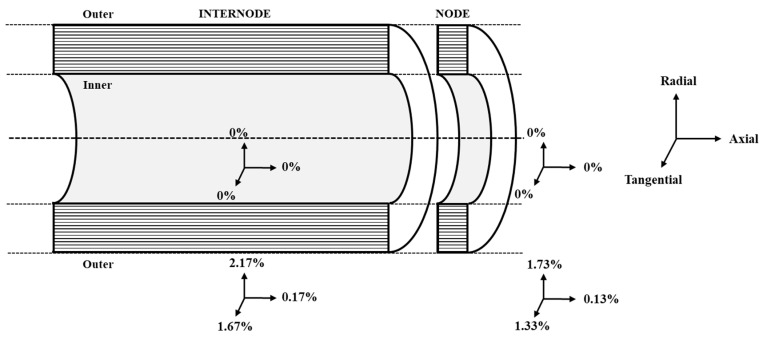
Schematic illustration of culm shrinkage model showing dimensional changes between inner and outer layers by considering an increasing exponential gradient of thermal expansion.

The data from previous studies, and as observed in experimental investigation in the previous section, showed that the shrinkage effect clearly varies along and across sections of bamboo culms as shown in Figure 8 [14,15,20]. In this investigation, an exponential shrinkage model in terms of variation in thermal contraction was assumed across the cross-section of bamboo, which is in line with the graded distribution of its vascular bundles [50].

**Figure 9:**
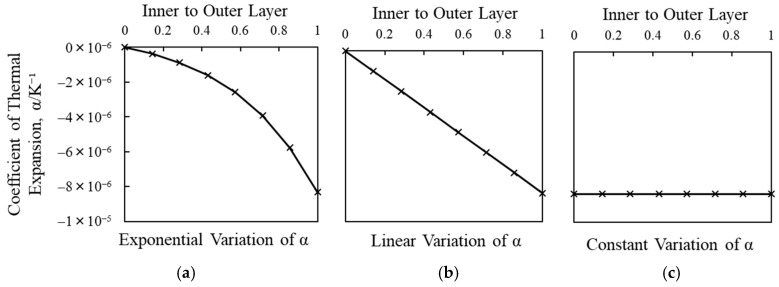
Three types of shrinkage models investigated: (**a**) exponential, (**b**) linear and (**c**) constant shrinkage models.

The coefficient of linear thermal expansion *α* was determined from the following expression:

(2) $$\left[\alpha =\;\frac{1}{L}\;\frac{\Delta L}{\Delta T}\right]$$

where *α* is the coefficient of thermal expansion in K⁻¹, *L* is the original length, Δ*L* is the change in length and Δ*T* represents the change in temperature. To confirm the hypothesis about the contribution of graded variation of thermal contraction (Figure 9a) across wall thickness of bamboo culm on its mechanisms of longitudinal split, two other shrinkage models were considered. They were linear and constant shrinkage models as shown in Figure 9b,c respectively.

### 4.2. Material Parameters

FEM simulation was conducted to determine the instant of crack initiation due to effect of thermal changes on shrinkage of bamboo culm. Based on the shrinkage experiment conducted in a 24- and 48-h period, the changes in dimensional values obtained were adjusted by considering a factor of 5 in order to simulate shrinkage behavior within the first few hours of treatment. The change in temperature for thermal modification was taken as 200 °C. The adjusted dimensional values and their corresponding coefficient of thermal expansion determined by Equation (2) are listed in Table 3.

Bamboo culm has a unique hollow morphology consisting of nodes and internodes which provide structural support to its uppermost section [32]. The shrinkage of the node section was assumed to be 25 percent lower than that of the internode section based on the observation made by Huang et al. (2018) in a previous study [14]. Here, the node, which has a structural significance by improving the lateral stability and stiffness of the culm structure, was assumed as the strongest part as was the case in previous studies [2,20,32]. Thus, it was modeled as a stiffer section by assigning with material data twice of that of the internode section as indicated in Table 4. The engineering constants of the orthotropic material parameters of the internode as indicated in Table 4 was taken from previous study whereby the modulus of elasticity in the longitudinal and transverse directions were determined as 15 and 0.675 GPa, respectively [32]. The Poisson’s ratio *υ*_L_ and *υ*_T_ in the longitudinal and transverse directions were determined as 0.3 and 0.0135, respectively from past literature data [28,32]. The shear modulus of the internode section was taken as 630 MPa from previous study [32].

### 4.3. Geometrical Modeling and Boundary Conditions

**Figure 10:**
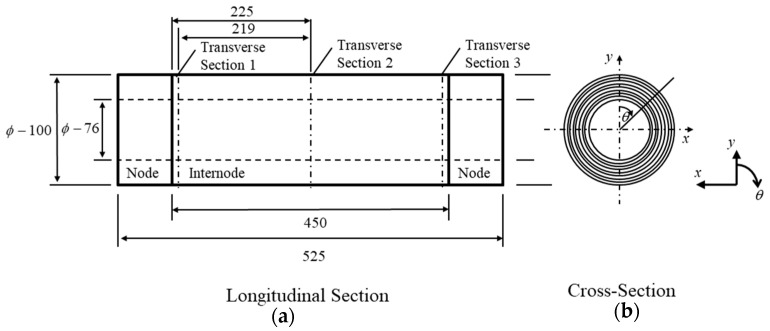
(**a**) Longitudinal section showing dimension outline of solid cylindrical model and (**b**) coordinate system in cross-sectional view.

The outer diameter, wall thickness and intermodal length of the physical model were taken as 100, 12 and 450 mm, respectively based on the morphological data of Madake bamboo with an average internode count of 18 [50]. To simulate the hollow bamboo structure, a cylindrical model with thick-walled section was used. The boundary conditions of the shrinkage simulation are shown in Figure 10. The effect of thermal contraction on shrinkage was simulated on LS-DYNA FEM software (Version R9.3.1, Livermore Software Technology, Livermore, CA, USA), by considering a thermal expansion material model.

### 4.4. FE Mesh

**Figure 11:**
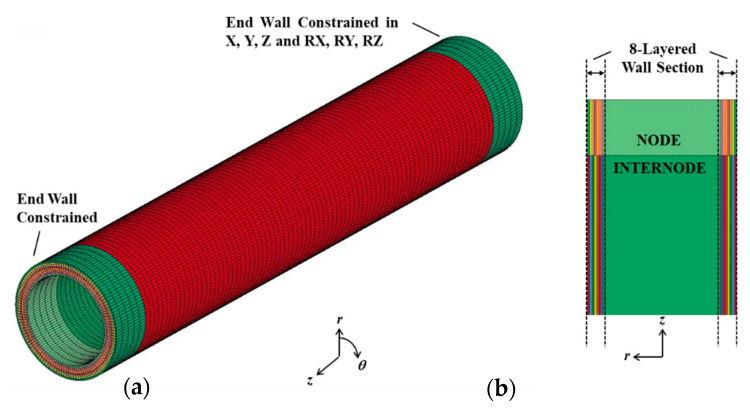
(**a**) Hexahedral mesh solid of internodal unit of bamboo model (number of elements: 89280, number of nodes: 101556) and (**b**) illustration of 8-layered wall section.

A finite element modeling and post processing (FEMAP) software (Siemens Digital Industries Software, Plano, TX, USA) was used to design the model. The meshed domain was discretized into 101556 nodes and 89280 elements by applying hexahedral mesh solid as displayed in Figure 11.

## 5. Numerical Results

### 5.1. Strain Field Analysis

**Figure 12:**
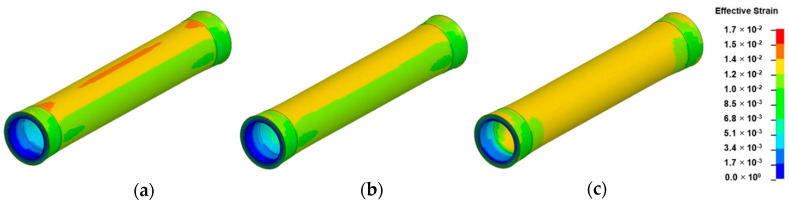
Fringe plots of effective strain of: (**a**) exponential; (**b**) linear and (**c**) constant shrinkage models in intermodal unit of bamboo culm due to effect of thermal contraction.

The variation of effective strain in bamboo culm model due to effect of thermal contraction is illustrated in Figure 12. The displayed models in Figure 12 were assigned with a displacement scale factor of 10 to emphasize on the noticeable shrinkage effect caused by thermal contraction. The contrast in fringe plots of Figure 12a–c occurred as a result of variation in gradient of thermal expansion coefficient. Distinct regions of strain concentration can be clearly discerned in Figure 12a,b. As demonstrated in Figure 12a, the distinctive narrowed region of strain concentration is in line with previous observation made about splitting along bamboo culm during shrinkage [51].

The uneven shrinkage across bamboo layers induced a restraining effect leading to build up of tensile forces in the outermost layers, hence giving rise to the region of strain concentration as observed in Figure 12a. In Figure 12c however, which is the control model with a constant thermal expansion coefficient across its wall thickness, a uniform distribution of effective strain was observed across its layers. Hence, it can be deduced that the existence of a gradient in thermal expansion across bamboo wall section affects its uniform contraction with increasing temperature.

### 5.2. Analysis of Effective Strain Distribution

**Figure 13:**
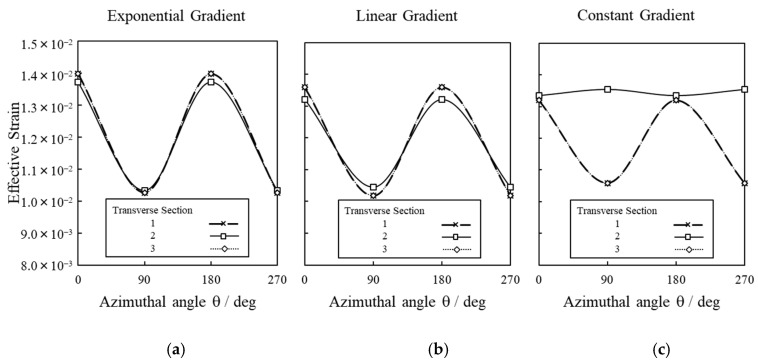
Distribution of effective strain in terms of azimuthal angle, θ at transverse Section 1, Section 2 and Section 3 in three types of shrinkage models corresponding to: (**a**) exponential gradient, (**b**) linear gradient and (**c**) constant gradient.

The shrinkage due to variation in gradient of thermal expansion coefficient is quantitatively assessed on analyzing the effective strain distribution in the outermost periphery of the intermodal culm-section. The distribution of effective strain in terms of azimuthal angle, θ, along the transverse sliced Section 1, Section 2 and Section 3 (Figure 10a) is represented in Figure 13. The azimuthal angle is taken as clockwise in reference to the vertical axis (Figure 10b).

The increase in peaks at azimuthal angle of 0° and 180° (Figure 13a–c) indicates the onset of fracture during shrinkage. Based on the results of Figure 13c, the effective strain distribution was confirmed to be constant around azimuthal angle *θ* at transverse Section 2. Thus, the constant thermal gradient across the wall thickness was assumed to reduce the propensity to cracking due to the absence of distinct area of strain concentration.

From Figure 13a,b, the similarity in the distribution of effective strain at transverse Section 2 in comparison to transverse Section 1 and Section 3, indicates distinct regions along full intermodal length, which are subjected to either compressive or tensile forces. The graded variation in thermal expansion coefficient can thus be associated with the inducement of internal forces, namely tensile and compressive forces, at specific regions along the culm length, which eventually leads to sudden split.

### 5.3. Analysis of Bamboo Fracture Mechanisms due to Thermal Contraction

Besides the non-linear increase in dimensional changes with increasing temperature, the notable difference which prevailed between radial-tangential directions in comparison to longitudinal one, significantly influenced its shrinkage behavior in the formerly mentioned directions. As observed from FTIR results, the principal cellular constituents of bamboo structure, namely cellulose, hemicellulose and lignin, which are accountable for its high mechanical strength, undergo major changes at elevated temperature between 150 °C and 200 °C. Shrinkage effect at elevated temperature was noticeable as a result of weight loss by reduction in weakly bound water and increase in desorption of water content. Besides, MC was reported to be further reduced as a result of thermal degradation of hemicellulose which also contributed to an increase in brittleness due to increase in arrangement of crystalline cellulose [52,53].

**Figure 14:**
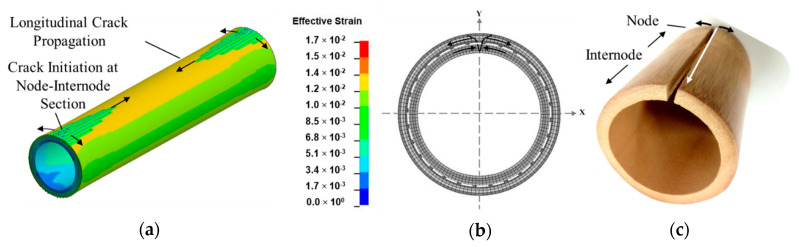
Failure mechanisms of bamboo due to shrinkage: (**a**) fringe plot of effective strain of internode section in exponential shrinkage model depicting crack initiation and propagation by implementing an element erosion technique; (**b**) node-internode transverse section showing layered innermost and outermost regions induced with compressive and tensile forces, respectively and (**c**) longitudinal split in natural bamboo.

The corresponding mechanisms associated with the effects of specific changes in the radial and tangential directions on the shrinkage behavior of bamboo was further explored by FEM by considering various shrinkage models. Based on the exponential shrinkage model, the extent of difference in terms of contraction which prevailed between the innermost and outermost layers as a result of non-uniform thermal gradients led to tensile stress build up in the latter as illustrated in Figure 14b. Both the outer shrank layers and nodes are assumed to promote residual stress build-up within the culm structure by exerting a restraining effect on its remaining section.

Figure 14a illustrates the failure mechanisms observed in bamboo culm due to effect of thermal contraction. The stages of crack initiation and propagation as depicted in Figure 14a were visualized by implementing an element erosion technique on LS-DYNA. Non-uniform shrinkage across its wall thickness induced compressive and tensile forces in the layered innermost and outermost regions, respectively as shown at the node-internode transverse section in Figure 14b. Hence, split due to shrinkage is assumed to originate from the outermost layer of the node-internode section of the culm structure as predicted in a previous investigation by Hone et al. [51]. This is assumed to be the critical location of crack initiation point which is preceded by the longitudinal propagation with minimum resistance along the intermodal length.

### 5.4. Future Recommendation

In addition to the variation in gradient of thermal expansion coefficient, another important factor not investigated in this study could significantly influence the instance of crack initiation: the rate of temperature increase. Additionally, as evidenced by FTIR results, the molecules responsible for color change in bamboo could be further investigated. Precise monitoring of color change could assist in prediction of an optimum treatment temperature without engendering irreversible changes by thermal degradation. Remote and non-destructive control of the actual chemical state and thus of the strength could also become possible upon monitoring the chromophore emission in real buildings.

## 6. Conclusions

Bamboo recurrently absorb or desorb moisture to attain an EMC as a result of its hygroscopic nature. For improved dimensional stability, heat treatment was found to be an effective measure in lowering the rate of water absorption; however, it came at the expense of non-uniform shrinkage and sudden split. From investigation of thermal modification, correlation between the dimensional changes in the three principal directions of bamboo and its orthotropic characteristic was established. Shrinkage in the radial and tangential directions significantly exceeded that in the longitudinal direction. Similar trend in terms of exponential change in weight decrement was observed with increasing temperature. From FTIR results, noticeable shrinkage occurred as result of weight loss by reduction in weakly bound water and increase in desorption of water content while alteration to its mechanical properties was attributed to changes in cellulose, hemicellulose, and lignin between temperature range of 150 °C to 200 °C.

Secondly, the effect of graded hierarchical structure on shrinkage behaviour of bamboo could be clearly discerned by considering variation in gradient of thermal expansion coefficient across its wall thickness in numerical simulations. The exponential thermal gradient model was found to yield a narrowed region of strain concentration, which is associated with the longitudinal crack propagation pathway along the intermodal length. From this investigation, it could be concluded that despite its beneficial trait of enhancing the dimensional stability of the culms, the corresponding effects of thermal modification on the highly orthotropic nature of bamboo can inadvertently trigger an adverse effect across its wall thickness thereby leading to split. Results of this study can be useful in order to achieve optimized durability during the modification stage of bamboo construction material as well as monitoring of its color change in response to alteration of its durability during its service lifetime.

## Figures and Tables

**Table 1:** Weight and dimensional changes of Moso bamboo specimens during thermal modification at three different temperatures (Mn and CV represent the mean and coefficient of variance, respectively).

Specimen	Temp/°C	Weight Loss/%				Radial Shrinkage/%				Tangential Shrinkage/%				Longitudinal Shrinkage/%			
		24 h		48 h		24 h		48 h		24 h		48 h		24 h		48 h	
		Mn	CV	Mn	CV	Mn	CV	Mn	CV	Mn	CV	Mn	CV	Mn	CV	Mn	CV
With Epidermis	100	3.3	0.03	3.3	0.03	0.7	0.31	1.1	0.10	1.0	0.17	1.2	0.13	0.2	0.82	0.3	0.43
	150	9.9	0.04	15	0.03	1.3	0.12	2.7	0.20	1.7	0.13	2.8	0.04	0.1	0.50	0.9	0.07
	200	41	0.03	50	0.03	17	0.03	23	0.01	18	0.08	23	0.12	0.6	0.52	1.1	0.10
Without Epidermis	100	3.4	0.01	3.4	0.01	0.6	0.35	0.7	0.30	0.9	0.14	1.1	0.16	0.3	0.65	0.4	0.74
	150	10.0	0.03	16	0.02	1.2	0.81	1.6	0.52	0.9	0.49	2.1	0.12	0.2	0.22	0.7	0.20
	200	41	0.03	51	0.02	18	0.18	24	0.06	18	0.09	25	0.06	0.1	0.91	1.1	0.10

**Table 2:** Characteristic bands of FTIR spectra of bamboo samples in the frequency interval of 400 to 3600 cm^−1^.

Peak	Assignment	Frequency (cm^−1^)	References
1	C-C deformation	423	
2	C-H deformation in cellulose	605	[40]
3	C-H deformation of glucose ring in cellulose and hemicellulose	897	[40]
4	C-O, C-H primary alcohol, guaiacyl (lignin)	1039	[40]
5	C-O-C Carbohydrate from hemicellulose	1160	[40,41]
6	Guaiacyl ring breathing with CO-stretching (lignin and hemicelluloses), esters	1242	[40,41,42]
7	C=O free carbonyl groups, Stretching of acetyl or carboxylic acid (hemicelluloses)	1730	[40,41,42,43,44,45]
8	Adsorbed CO_2_	2317, 2351, 2372	[46]
9	C-H in Cellulose and hemicellulose stretching	2945	[41,45,47]
10	O-H stretching in Alcohols, phenols, acids, and weakly bounded absorbed water from lignin.	3400	[41,45,47]
11	O-H stretching of adsorbed water and intermolecular bonded OH.	3566, 3597, 3618	[46]

**Table 3:** Thermal expansion parameters used in FEM simulation.

Thermal Expansion Parameters						
Parameter	Internode			Node		
	Radial	Tangential	Longitudinal	Radial	Tangential	Longitudinal
Change in Length Δ*L* (%)	2.17	1.67	0.17	1.73	1.33	0.13
Coefficient of Thermal Expansion α (K⁻¹)	−1.08 × 10^−4^	−8.33 × 10^−5^	−8.33 × 10^−6^	−8.67 × 10^−5^	−6.67 × 10^−5^	−6.67 × 10^−6^

**Table 4:** Orthotropic material parameters used in FEM simulation.

Orthotropic Material Parameters									
Internode					Node				
Elastic Modulus (MPa)		Poisson’s Ratio		Shear Modulus (MPa)	Elastic Modulus (MPa)		Poisson’s Ratio		Shear Modulus (MPa)
*E*_L_	*E*_T_	*ν*_L_	*ν*_T_	*G*_L_	*E*_L_	*E*_T_	*ν*_L_	*ν*_T_	*G*_L_
15,000	675	0.3	0.0135	630	30,000	1350	0.3	0.0135	1260

## Data Availability

The data presented in this study are available on request from the corresponding author. The data are not publicly available due to private restrictions.
